# Optimizing the clinical assessment of pseudoprogression in patients with solid tumors treated with checkpoint inhibitors: a systematic literature review and meta-analysis of associated features

**DOI:** 10.1007/s00262-026-04433-9

**Published:** 2026-06-08

**Authors:** Georgios Fountoukidis, Elisabet Tina, Anna Göthlin-Eremo, Gustav Ullenhag, Antonios Valachis

**Affiliations:** 1 Department of Oncology, Faculty of Medicine and Health, Örebro University, SE 70182 Örebro, Sweden; 2 Department of Clinical Research Centre, Faculty of Medicine and Health, Örebro University, SE 701 82 Örebro, Sweden; 3 Department of Oncology, Uppsala University Hospital, Entrance 101, First Level, 751 85 Uppsala, Sweden; 4 Department of Immunology, Genetics and Pathology, Science for Life Laboratory, Uppsala University, Uppsala, Sweden

**Keywords:** Pseudoprogression, Immune checkpoint inhibitors, iRECIST, Metastatic solid tumors, Meta-analysis

## Abstract

**Background:**

Pseudoprogression is an atypical response pattern observed during treatment with immune checkpoint inhibitors (CPIs) that can complicate clinical decision-making. It is characterized by initial radiologic progression followed by subsequent tumor regression or stabilization, with confirmation requiring repeat imaging. We aimed to characterize the clinical and imaging features of confirmed pseudoprogression in solid tumors treated with CPIs using pooled study data.

**Methods:**

We systematically searched MEDLINE, Embase, and Web of Science through December 2025. Prospective and retrospective cohort studies and randomized trials reporting confirmed pseudoprogression during CPI therapy were eligible. Two independent reviewers performed study selection and data extraction using predefined criteria. Study quality was assessed using the Joanna Briggs Institute checklist. Pooled analyses were conducted with RevMan and Stata. Outcomes included time to initial radiologic progression, magnitude of tumor burden increase, occurrence of new lesions, changes in nontarget lesions, and subsequent objective response.

**Results:**

Thirteen retrospective studies were included; most applied iRECIST criteria. The pooled median time to initial progression was 2.52 months (95% CI, 1.54–3.51). Mean tumor burden increase was 33.0% (95% CI, 22.7–43.3), and new lesions occurred in 35.3% of cases. Tumor burden increases > 100% were rare (3.9%). Despite initial progression, 41.8% subsequently achieved partial response and 6.4% complete response without therapy change. Median time to best response was 2.79 months (95% CI, 0.62–7.20).

**Conclusions:**

Pseudoprogression typically occurs early during CPI therapy and is associated with modest tumor growth. Marked tumor increases are uncommon and may help distinguish true progression. Careful clinical and radiologic assessment remains essential.

**Supplementary Information:**

The online version contains supplementary material available at 10.1007/s00262-026-04433-9.

## Introduction

In recent years, immunotherapy with checkpoint inhibitors (CPIs) has represented a major advancement in cancer treatment, leading to long-term remissions and extended survival [1, 2]. However, integrating immunotherapy into clinical practice poses several challenges, including the occurrence of atypical response patterns that differ from those typically observed with most conventional treatments such as chemotherapy [1–4].

One challenging atypical response is pseudoprogression, characterized by initial radiologic evidence of disease progression followed by subsequent tumor regression or stabilization on repeat imaging. The reported frequency of pseudoprogression during immune checkpoint inhibitor therapy ranges from 6 to 10%, depending on the response criteria applied [1–5]. Current radiologic guidelines, such as immune Response Criteria In Solid Tumours (iRECIST), require two consecutive imaging assessments to distinguish true progression from pseudoprogression [1, 6, 7]. At present, no validated clinical or biological markers exist to diagnose pseudoprogression at its first appearance. Sometimes, pseudoprogression can be ruled out due to clear clinical progressive disease, but the diagnosis can typically be confirmed by repeat imaging, although in selected cases histopathological confirmation may be possible.

This uncertainty carries important clinical consequences as prematurely discontinuing an effective therapy in cases of pseudoprogression may deny patients a potential benefit, whereas continuing treatment in case of true progression may expose them to unnecessary toxicity and delay alternative treatment strategies. Therefore, early and accurate differentiation between these two scenarios is critical.

Previous systematic reviews and meta-analyses have examined pseudoprogression in specific contexts as in lung cancer, as well as across different malignancies, primarily focusing on incidence, response patterns, and outcomes. However, the clinical and imaging features associated with confirmed pseudoprogression have not been comprehensively synthesized [8, 9]. Improved characterization of these features may help clinicians better distinguish pseudoprogression from true progression early in the treatment course.

The aim of this study was to explore clinical and imaging factors associated with pseudoprogression by systematically pooling data from published studies describing patients with confirmed pseudoprogression.

We conducted a systematic literature review and meta-analysis of published studies reporting detailed clinical and/or imaging characteristics of pseudoprogression in patients with metastatic solid tumors treated with CPIs.

## Methods

The study protocol was prospectively registered in the PROSPERO database (Registration number: CRD42024618195). The reporting of this review follows the PRISMA (Preferred Reporting Items for Systematic Reviews and Meta-Analyses) guidelines [10].

### Search strategy

We systematically searched three electronic databases (MEDLINE, Web of Science, and Embase) without restrictions on publication year or language. The last search update was conducted in December 2025. The complete search algorithm is provided in Supplementary Table 1.

### Study selection process

Two independent reviewers (GF and AV) screened all search results to identify potentially eligible studies. Studies were considered eligible if they met the following criteria: (a) reported detailed clinical and/or imaging data on pseudoprogression patterns in patients with metastatic solid tumors; (b) included patients treated with any type of CPI; and (c) included at least seven patients with confirmed pseudoprogression to allow for relevant pooled analyses. This threshold was applied to exclude very small case series and reduce small sample bias, thereby ensuring more stable pooled estimates. Any definition of pseudoprogression as described by each primary study was accepted.

We excluded studies that did not report sufficient data on at least one of the predefined clinical and/or imaging data on pseudoprogression patterns (timing of progression, tumor burden changes, or response after pseudoprogression), patients with hematologic malignancies, and patients receiving CPI in combination with other systemic therapies administered concurrently. The use of concurrent palliative radiotherapy was not consistently reported and could not be reliably assessed. After the initial screening, full texts of potentially eligible studies were reviewed by the same two researchers (GF and AV) to confirm eligibility. Discrepancies were resolved through discussion until consensus was reached.

### Data extraction and quality assessment

Two reviewers (GF and AV) independently extracted data from the included studies using a standardized data collection form. The following variables were collected: study characteristics (first author, journal, year of publication, country of origin, study design, and inclusion period); patient and treatment characteristics (cancer type, type of CPI used, age, sex, duration of treatment until pseudoprogression, and best response after pseudoprogression [complete response, partial response, or stable disease]); pseudoprogression definition; and imaging-related factors (baseline tumor burden, tumor burden at pseudoprogression, increase in nontarget lesions according to RECIST at pseudoprogression, and occurrence of new lesions at pseudoprogression).

The quality of each included study was assessed independently by both reviewers using the Joanna Briggs Institute (JBI) Checklist for Case Series. Any disagreements were resolved through discussion.

### Data synthesis

Statistical analyses were conducted by pooling outcomes across primary studies. For proportion-based endpoints, raw numerators and denominators were extracted to calculate study-level proportions, which were then combined to generate pooled estimates with corresponding 95% confidence intervals (CI). For continuous outcomes related to time-to-event measures or percentage increases in lesions, study-level means and standard deviations (SD) were used; when SDs were not reported, they were derived from available summary statistics following established methods. All meta-analyses were performed using random-effects models to account for the small sample sizes and the anticipated clinical heterogeneity across studies. Statistical heterogeneity was quantified using the *I*^2^ statistic. Analyses were conducted using Statsdirect (version 4.0.4, StatsDirect statistical software. England: StatsDirect Ltd 2024) and Stata (version 17; StataCorp, College Station, TX, USA).

All steps of the systematic literature review conducted using the Covidence (Covidence systematic review software, Veritas Health Innovation, Melbourne, Australia). The study was registered prospectively in the open database PROSPERO (CRD42024618195).

## Results

### Characteristics of eligible studies and patients

**Fig. 1:**
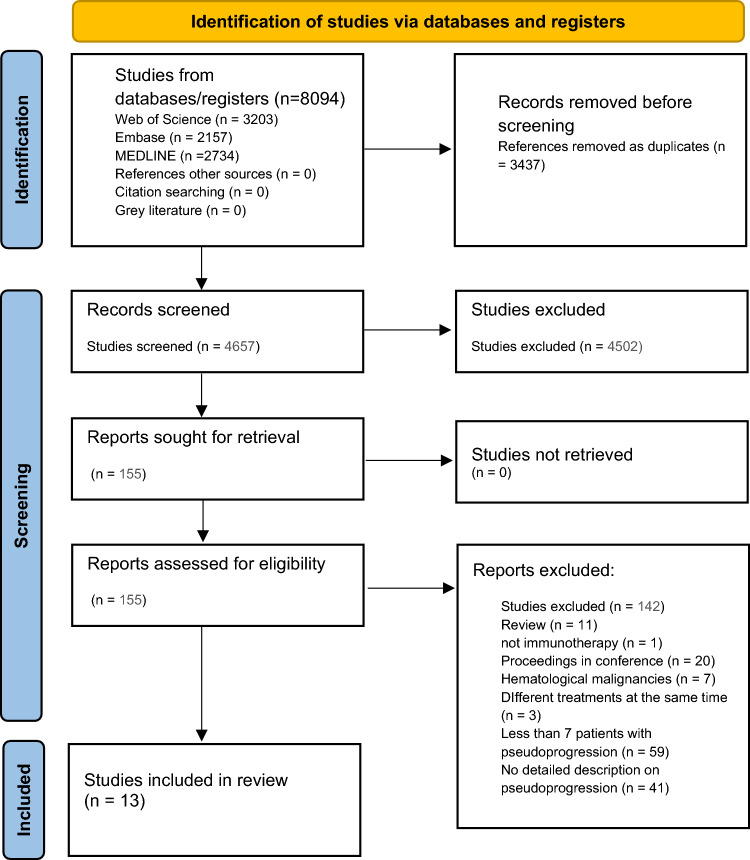
Flowchart illustrating the identification, screening, eligibility assessment, and inclusion of studies in the systematic review

After applying all inclusion and exclusion criteria to the 4,657 studies initially identified, 13 studies were included in the final meta-analysis [3, 9, 11, 21]. The study selection process is illustrated in Fig. 1.

The main characteristics of the included studies and patient populations are summarized in Table 1. Given the heterogeneity and variability in reporting across studies, pooled demographic estimates were not considered appropriate. Of the 13 studies, nine were retrospective cohort studies [3, 9, 11, 12, 15–17, 20, 21], while four were retrospective analyses of single-arm clinical trials [13, 14, 18, 19].

**Table 1:** Characteristics of eligible studies and patients

Author (ref)	Study type	Inclusion period	Definition of pseudoprogression	N patients with pseudoprogression	Age Median	Sex (F/M)	Type of malignancy	Type of checkpoint inhibitor
Ahmed [11]	Retrospective	Nov 2023–Nov 2024	iRECIST	14	NR	NR	Melanoma	Pembrolizumab
Basler [12]	Retrospective	2013–2019	PP was defined as a diameter increase by ≥ 20% at TP1, followed by a decrease to < 20% at TP2 compared with TP0	9	74 (IQR: 64–78)	3/6	Melanoma	PD-1 inhibitors as monotherapy (n = 7) or combined immunotherapy (n = 2)
Beaver [13]	Retrospective analysis of single-arm trials	NR	RECIST-defined response after RECIST-defined progression	57	62 (IQR: 52–68)	20/37	Melanoma	PD-1 inhibitors as monotherapy
Bernard‑Tessier [14]	Retrospective analysis of single-arm trial	Sept 2015–Nov 2017	iRECIST	10	59.7 (range: 33.9–74.7)	3/7	Various	NR
Colle [15]	Retrospective	Feb 2015–Dec 2019	iRECIST	12	53.5 (IQR: 45–65)	4/8	Gastrointestinal	PD-1 inhibitors as monotherapy (n = 9) or combined immunotherapy (n = 3)
Da Silva [19]	Retrospective analysis of single-arm trial	Dec 2013–Mar 2018	RECIST-defined response after RECIST-defined progression	13	NR	NR	Melanoma	Nivolumab + ipilimumab
Fujimoto [16]	Retrospective	Jan 2016–Dec 2016	RECIST-defined response after RECIST-defined progression	14	68.5 (IQR: 63–72)	2/12	NSCLC	Nivolumab
Haaker [17]	Retrospective	Nov 2012–Jul 2022	iRECIST	8	70 (range: 51–86)	3/5	Renal cell carcinoma	Nivolumab
Hodi [18]	Retrospective analysis of single-arm trial	NR	irRC and RECIST v1.1	15	NR	NR	Melanoma	Pembrolizumab
Monch [3]	Retrospective	NR	iRECIST	32	67 (IQR: 62.5–76.5)	7/25	Various	Various
Morrissey [9]	Retrospective	May 2006–Oct 2022	iRECIST	9	50	NR	Various	NR
Tazdait [20]	Retrospective	Feb 2013–Oct 2016	iRECIST	8	NR	NR	NSCLC	PD-1 inhibitors as monotherapy
Won [21]	Retrospective	Mar 2016–Jul 2018	Progressive disease on iRECIST or RECIST1.1 and subsequently reset to non-PD categories	7	NR	NR	NSCLC	NR

*Abbreviations*: F/M, Female/male; NR, Not reported; IQR, Interquartile range; NSCLC, Non-small cell lung cancer; PP, Pseudoprogression; TP0, Baseline; TP1, First follow-up at 3 months; TP2, Second follow-up at 6 months

Table 1 summarizes the design characteristics and patient demographics of the studies included in the analysis. For each study, the study type, inclusion period, definition of pseudoprogression, number of patients with pseudoprogression, age distribution, sex, cancer type, and immune checkpoint inhibitor regimen are reported

The most common tumor type represented was melanoma (six studies), followed by non-small cell lung cancer (NSCLC) (three studies). The number of patients with confirmed pseudoprogression per study ranged from 7 to 57.

Regarding the definition of pseudoprogression, eight studies used iRECIST criteria [9, 11, 14, 15, 17, 20, 21], while the remaining studies relied on alternative or study-specific definitions [9, 10, 13, 15, 17].

### Quality assessment of included studies

Quality assessment using the JBI Checklist for Case Series indicated that most studies adequately addressed the key quality domains. However, several studies had unclear inclusion criteria, inconsistent outcome measurements, or incomplete demographic and clinical data (Supplementry Table 2).

### Features related to pseudoprogression

The pooled analyses of clinical and imaging features associated with pseudoprogression are presented in Table 2. The pooled median time from CPI initiation to pseudoprogression was 2.52 months (95% CI 1.54–3.51 months; *I*^2^: 91.9%). At the time of pseudoprogression, the mean percentage increase in tumor burden was 33.0% (95% CI 22.7–43.3%; *I*^2^: 46.6%), with 3.9% of cases (95% CI 1.4–7.8%; *I*^2^: 45.1%) showing an increase in tumor burden of greater than 100% from baseline. New lesions at the time of pseudoprogression were observed in 35.3% of patients (95% CI 17.6–55.4%; *I*^2^: 84.2%), whereas nontarget lesion growth occurred in 22.0% of cases (95% CI 9.1–38.4%; *I*^2^: 76.1%).

**Table 2:** Pooled analyses of pseudoprogression-related features

Feature	Number of studies	Number of patients	Pooled effect size (95% Confidence Interval)	*I*^2^
Time until initial progressive disease, pooled mean in months	8	127	2.52 (1.54–3.51)	91.9
% increase of lesions from baseline, pooled mean in % > 100% increase of lesions from baseline, pooled proportion (%)	7 7	143 143	33.0 (22.7–43.3) 3.9 (1.4–7.8)	46.6 45.1
Increase in nontarget lesions, pooled proportion (%)	7	143	22.0 (9.1–38.4)	76.1
New lesions at initial progression, pooled proportion (%)	9	164	35.3 (17.6–55.4)	84.2
Response patterns after pseudoprogression (%) Complete response, pooled proportion Partial response, pooled proportion Time until best response, pooled mean in months	8 8 4	153 153 88	6.4 (2.0–13.2) 41.8 (29.9–54.1) 2.79 (0.62–7.20)	45.5 51.9 85.5

Table 2 presents pooled estimates of radiological features and response patterns associated with pseudoprogression, reported as pooled means with 95% confidence intervals (CIs). Between-study heterogeneity was assessed using the *I*^2^ statistic, representing the proportion of total variance attributable to heterogeneity rather than chance, with higher values indicating greater heterogeneity

### Treatment responses after pseudoprogression

Following pseudoprogression, partial response was achieved in 41.8% of patients (95% CI 29.9–54.1%; *I*^2^: 45.5%), whereas complete response was observed in 6.4% (95% CI 2.0–13.2%; *I*^2^: 51.9%). The pooled median time from pseudoprogression to best response was 2.79 months (95% CI 0.62–7.20 months; *I*^2^: 85.5%) (Table 2).

## Discussion

Through an extensive evaluation of confirmed cases of pseudoprogression, this systematic review and meta-analysis provide important insights into the clinical and radiologic features of pseudoprogression in patients with metastatic solid tumors treated CPI. By pooling data across published studies, we identified consistent patterns that can help clinicians better recognize this phenomenon and distinguish it from true progression.

Our findings indicate that pseudoprogression typically occurs early during treatment, with a pooled median time of 2.5 months from CPI initiation to the first radiologic evidence of progression. This time frame is consistent with the median time of 8 weeks reported in a pooled analysis of an institutional case series and published case reports involving patients with lung cancer treated with CPI [8]. This early timing underscores the need for enhanced surveillance during the initial phases of immunotherapy, when atypical response patterns are most likely to emerge. At the time of pseudoprogression, we observed a median increase in tumor burden of 33%, with most cases showing less than a 50% increase compared to baseline. Importantly, increases greater than 100% were rare, suggesting that very large tumor growth is unlikely to represent pseudoprogression and may be used as a practical threshold in clinical decision-making.

We also found that new lesions were present in approximately one-third of patients, while progression of nontarget lesions occurred in one-fifth of cases. These findings are clinically relevant because they demonstrate that both new lesions and increase in nontarget lesion can be part of a pseudoprogression pattern and should not automatically be interpreted as treatment failure. This aligns with prior reports from pooled analyses of clinical trials in which mixed responses and immune-related inflammatory changes were described as common during CPI therapy [9]. These data support a more nuanced interpretation of imaging findings, in which radiologists and oncologists carefully evaluate the whole disease burden, rather than focusing solely on target lesions.

Following pseudoprogression, a substantial proportion of patients experienced clinically meaningful tumor regression. An objective response was achieved in nearly half of the patients, with 41.8% experiencing partial response and 6.4% complete response. The median time from pseudoprogression to best response was 2.8 months, indicating that in clinically stable patients, continuing treatment beyond initial radiologic progression may be justified. These findings are consistent with the previously reported pooled analysis of case series and case reports in lung cancer patients [8] and support the rationale behind immune-specific response criteria such as iRECIST, which recommend confirmatory imaging before labeling progression as definitive [6, 7].

Together, these data emphasize that early discontinuation of therapy based solely on radiologic findings may deprive patients of potential benefit, particularly in the absence of signs of clinical deterioration.

From a practical perspective, our results can help guide clinical decision-making when apparent progression is observed. First, the degree of tumor burden increase should be carefully considered. While small and moderate increases (≤ 50%) may represent pseudoprogression, tumor growth greater than 100% is rarely compatible with this phenomenon and strongly suggests true progression. Second, the appearance of new lesions or nontarget lesion growth should not automatically prompt treatment discontinuation, as these features are common in pseudoprogression. Instead, these findings should be interpreted in the context of the patient’s overall clinical status and correlated with other information, such as laboratory data including emerging biomarkers when available. Interestingly, circulating tumor DNA (ctDNA) dynamics have been suggested as potential predictive biomarkers for pseudoprogression, as they may provide a quantitative measure of tumor dynamics and help predict treatment response earlier than conventional imaging. However, their clinical use is currently limited by factors such as cost, turnaround time, and lack of standardization. Advanced imaging approaches, including radiomics, may also help differentiate pseudoprogression from true progression by capturing underlying biological differences. Pseudoprogression is thought to reflect immune cell infiltration and inflammatory changes, resulting in greater lesion heterogeneity, whereas in true progression a lesion more often consists of tumor cells. These differences may be detectable through quantitative imaging features across serial time points; however, such approaches remain investigational and require prospective validation before clinical implementation [12, 22, 23].

This study has several limitations. The number of eligible studies was relatively small, which limits the robustness and generalizability of our findings. A substantial degree of heterogeneity was observed across several analyses, likely reflecting differences in tumor types, study designs, and definitions of pseudoprogression. While some studies applied iRECIST, others used alternative or study-specific criteria, which may have introduced inconsistencies in patient classification and contributed to variability in the pooled estimates. Given the limited number of studies and the inconsistent reporting of key variables, more advanced analyses such as meta-regression were not considered statistically robust.

Despite these limitations, our meta-analysis provides a valuable foundation for identifying clinical surrogates of pseudoprogression that can support early assessment and guide clinical decision-making. Based on our findings, several key points should be considered: (1) new lesions and nontarget lesion growth are common and should prompt a comprehensive, whole-body assessment rather than immediate discontinuation of therapy; (2) pseudoprogression increases in tumor volume are typically around 30%, with most cases below 50%, while increases above 100% are rarely compatible with pseudoprogression; and (3) as objective responses can occur after pseudoprogression, careful evaluation and continuation of treatment in clinically stable patients are essential, while also informing patients about the possibility of atypical response patterns.

Further prospective studies are needed to more precisely characterize the clinical and imaging features of pseudoprogression and to validate these surrogates in diverse tumor types and treatment settings. Such research will help refine diagnostic criteria, improve early identification, and optimize management strategies for patients receiving CPI.

## Supplementary Information

Below is the link to the electronic supplementary material.

Supplementary file1 (DOCX 43 kb)

Supplementary file2 (DOCX 19 kb)

## Data Availability

All data supporting the findings of this study are derived from previously published articles and are included within the manuscript. No new datasets were generated.
