# Supplementary material for: Optimizing the clinical assessment of pseudoprogression in patients with solid tumors treated with checkpoint inhibitors: a systematic literature review and meta-analysis of associated features
Source: Cancer Immunol Immunother. 2026 Jun 8;75(6):170. doi: 10.1007/s00262-026-04433-9 (PMC13264642; doi:10.1007/s00262-026-04433-9)
Supplement: Supplementary file 1 — Supplementary file1 (DOCX 43 kb) [file 262_2026_4433_MOESM1_ESM.docx]

| Study (ref) | **Items of JBI critical appraisal tool** | | | | | | | | | |
| --- | --- | --- | --- | --- | --- | --- | --- | --- | --- | --- |
|  | **Criteria for inclusion** | **Reliable measurement of pseudoprogression** | **Valid identification pseudoprorgession** | **Consecutive patients** | **Complete inclusion** | **Reporting of demographics** | **Reporting of clinical information** | **Reporting of outcomes** | **Reporting of study sites** | **Appropriate statistical analysis** |
| Ahmed (11) | Yes | Yes | Yes | Yes | No | No | No | Yes | Yes | Yes |
| Basler (12) | Yes | Yes | Yes | Yes | No | Yes | Yes | Yes | Yes | Yes |
| Beaver (13) | Yes | Yes | Yes | No | Yes | Yes | Yes | Yes | Yes | Yes |
| Bernard‑Tessier (14) | Yes | Yes | Yes | Yes | Yes | Yes | Yes | Yes | Yes | Yes |
| Colle (15) | Yes | Yes | Yes | Yes | Yes | Yes | Yes | Yes | Yes | Yes |
| Da Silva (19) | Yes | Yes | Yes | Yes | Yes | Yes | Yes | Yes | Yes | Yes |
| Fujimoto (16) | Yes | Yes | Yes | Yes | Yes | Yes | Yes | Yes | Yes | Yes |
| Haaker (17) | Yes | Yes | Yes | Yes | Yes | Yes | Yes | Yes | Yes | Yes |
| Hodi(18) | Yes | Yes | Yes | Unclear | Unclear | No | No | Yes | Yes | Yes |
| Monch (3) | Yes | Yes | Yes | Unclear | Yes | Yes | Yes | Yes | Yes | Yes |
| Morrissey (9) | Yes | Yes | Yes | Yes | Yes | Yes | Yes | Yes | Yes | Yes |
| Tazdait(20) | Yes | Yes | Yes | Yes | Yes | Yes | Yes | Yes | Yes | Yes |
| Won (21) | Yes | Yes | Yes | Yes | Yes | Yes | Yes | Yes | Yes | Yes |

**Table 2. Quality assessment of included studies using the Joanna Briggs Institute (JBI) Critical Appraisal Checklist for Case Series.**
Each study was evaluated across predefined methodological domains. “Yes”, “No”, or “Unclear” indicate whether the criterion was met, not met, or insufficiently reported
