# Supplementary material for: Optimizing the clinical assessment of pseudoprogression in patients with solid tumors treated with checkpoint inhibitors: a systematic literature review and meta-analysis of associated features
Source: Cancer Immunol Immunother. 2026 Jun 8;75(6):170. doi: 10.1007/s00262-026-04433-9 (PMC13264642; doi:10.1007/s00262-026-04433-9)
Supplement: Supplementary file 2 — Supplementary file2 (DOCX 19 kb) [file 262_2026_4433_MOESM2_ESM.docx]

**Supplementary Table 1**

All searches were run on December 04, 2025.

Ovid MEDLINE(R) ALL 1946 toDecember 04, 2025

| Searchterms | | | Results |
| --- | --- | --- | --- |
| Pseudoprogression | | | |
|  | #1 | ("pseudoprogress*" or "Pseudo-progress*").ab,kf,ti. | 1393 |
|  | #2 | "atypical respons*".ab,kf,ti. | 1221 |
|  | #3 | "false progress*".ab,kf,ti. | 8 |
|  | #4 | "therapy-related change*".ab,kf,ti. | 104 |
|  | #5 | ("true progress*" or "true tumour progress*" or "true tumor progress*").ab,kf,ti. | 385 |
|  | #6 | 1 or 2 or 3 or 4 or 5 | 2833 |
|  | #7 | limit 6 to english language | 2734 |

Web of Science Core Collection (Clarivate)

(1975-present) Indexes=SCI-EXPANDED, SSCI, A&HCI, CPCI-S, CPCI-SSH, ESCI Timespan=All years

| Searchterms | | | Results |
| --- | --- | --- | --- |
| Pseudoprogression | | | |
|  | #1 | TS=("pseudoprogress*" OR "pseudo-progress*") | 2180 |
|  | #2 | TS=("atypical respons*") | 468 |
|  | #3 | TS=("false progress*") | 262 |
|  | #4 | TS=("therapy-related change*") | 196 |
|  | #5 | TS=("true progress*" OR "true tumour progress*" OR "true tumor progress*") | 838 |
|  | #6 | #1 OR #2 OR #3 OR #4 OR #5 | 3296 |
|  | #7 | (#1 OR #2 OR #3 OR #4 OR #5) AND LA=(English) | 3203 |

Embase.com (Elsevier)

| Searchterms | | | Results |
| --- | --- | --- | --- |
| Pseudoprogression | | | |
|  | #1 | 'pseudoprogression'/de | 167 |
|  | #2 | 'pseudoprogress*':ab,kw,ti OR 'pseudo-progress*':ab,kw,ti | 2730 |
|  | #3 | 'atypical respons*':ab,kw,ti | 598 |
|  | #4 | 'false progress*':ab,kw,ti | 17 |
|  | #5 | 'therapy-related change*':ab,kw,ti | 164 |
|  | #6 | 'true progress*':ab,kw,ti OR 'true tumour progress*':ab,kw,ti OR 'true tumor progress*':ab,kw,ti | 747 |
|  | #7 | #1 OR #2 OR #3 OR #4 OR #5 OR #6 | 3744 |
|  | #8 | #7 NOT 'conference abstract'/it | 2298 |
|  | #9 | #7 NOT 'conference abstract'/it AND [english]/lim | 2157 |

**The search strategy was designed to retrieve any references that mention pseudo-progression, or articles comparing true progress to false progress.** The rationale for this approach, rather than the conventional approach of searching for a combination of Population and Exposure, is that in this case the exposure comprises a multitude of factors that are not easily defined. The population poses a similar problem, as it includes a wide range of solid tumours treated with various checkpoint inhibitors. Therefore, we opted for a search strategy designed to retrieve any references mentioning terms related to false or true progression, regardless of population or exposure. A potential limitation of this approach is that articles that address pseudoprogression in the text body, without mentioning it in the title or abstract, may not be retrieved. However, in the absence of any reference to pseudoprogression in the title or abstract, sucharticles would likelynot have been identified as relevant during the screening process.
